# Haematogenous Spread of *Staphylococcus aureus* from an Iliacus Abscess to an ACL Reconstructed Knee

**DOI:** 10.1155/2013/914329

**Published:** 2013-05-16

**Authors:** Vivek Eranki, Andrew Wallis, Simon Smith

**Affiliations:** ^1^Royal Perth Hospital, 197 Wellington street, Perth, WA 6000, Australia; ^2^South West Health Campus, P.O. Box 5301, Bunbury, WA 6231, Australia

## Abstract

We describe a case of a 19-year-old male who presented to the South West Health Service with a septic knee, secondary to haematogenous spread from an iliacus abscess. Thus far, there have been no reported cases of haematogenous spread of infection from an iliacus abscess to an ACL reconstructed knee, let alone in a healthy young person with no risk factors. The patient has had several washouts of the knee along with the drainage of the abscess. The ACL graft was saved with the patient making a complete recovery.

## 1. Introduction

We describe a case of a 19-year-old male presenting with a septic knee, secondary to haematogenous spread from an iliacus abscess. The patient (Mr. P.) underwent an arthroscopic reconstruction of his ACL with a hamstring graft and extra articular fixation 4 months prior to this presentation. 

Mr. P. first presented via his GP to Busselton Hospital (rural centre) complaining of feeling unwell. He described an 8-day history of pain commencing in his lower back region and eventually involving the left knee. Working on a differential diagnosis of a rheumatologic illness, Busselton Hospital administered 100 mg of prednisolone over 48 hours. Subsequently, the patient received a single dose of ceftriaxone 2 g intravenously prior to aspiration of his left knee. The aspirate drained 86 mL of “yellow fluid.” He was then started on flucloxacillin 1 g QID intravenously. Microscopy on the aspirate showed large quantities of leukocytes and abundant growth of *Staphylococcus aureus* sensitive to flucloxacillin. His clinical picture worsened, and he was referred to orthopaedics at Bunbury Regional Hospital with rigors and spiking temperatures. 

**Figure 1:**
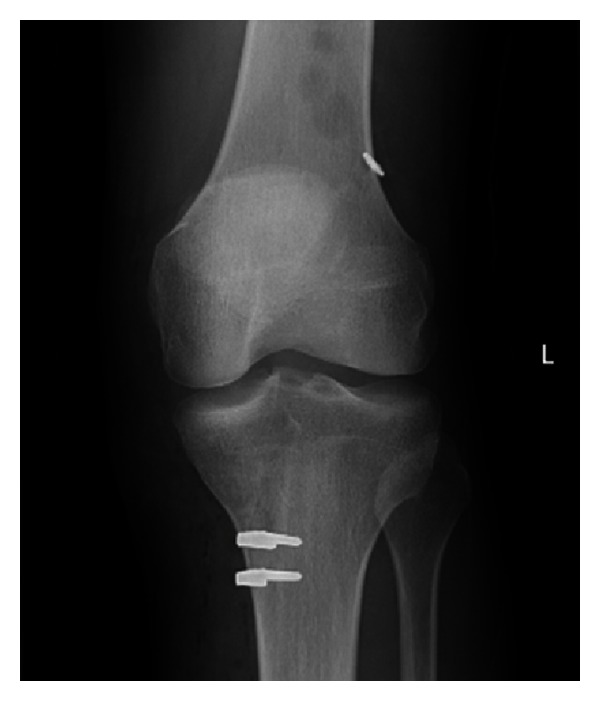
X-ray of the septic ACL reconstructed knee.

On admission, Mr. P. was unable to weight bear. He was haemodynamically stable and afebrile. His left knee was swollen, warm, and markedly tender. He had active flexion to 40 degrees and extension to 5 degrees, limited by pain and swelling. It was tender over his left sacroiliac joint, bilateral hips and right knee. Examination was otherwise unremarkable. His blood makers of infection were elevated with CRP reaching 318 mg/L and white cell count 15.8 × 10^9^/L (neutrophils 14.1 × 10^9^/L). Blood cultures showed *S. aureus* sensitive to flucloxacillin positive growth in two out of four bottles (one anaerobic and one aerobic). X-ray of the knee (Figure 1) showed moderate joint effusion within the suprapatellar recess. Besides recent knee surgery (4 months after ACL reconstruction), Mr. P. had no other risk factors for septic arthritis. He is a social smoker and consumes fewer than 2 standard drinks of alcohol per week. He denied drug use (intravenous or otherwise) and had no history of recent travel, tattoos, or sexual partners.

**Figure 2:**
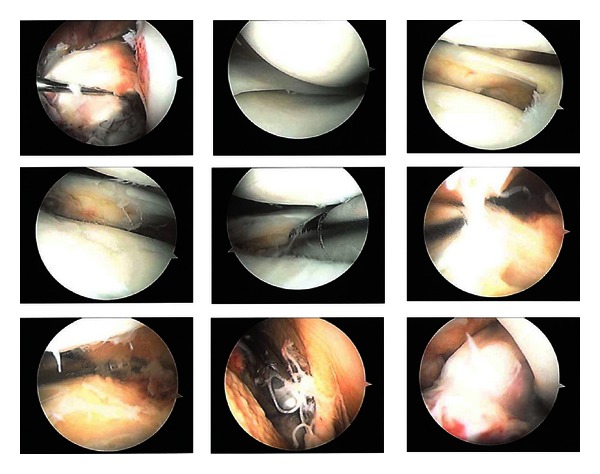
Knee arthroscopy.

Mr. P. underwent an arthroscopic washout of his left knee on the night of his presentation. The arthroscopy revealed frank pus within the left knee and an intact ACL graft with no evidence of infection or failure (Figure 2). Microbiology testing of the intraoperative specimen grew *S. aureus* sensitive to flucloxacillin. 

**Figure 3:**
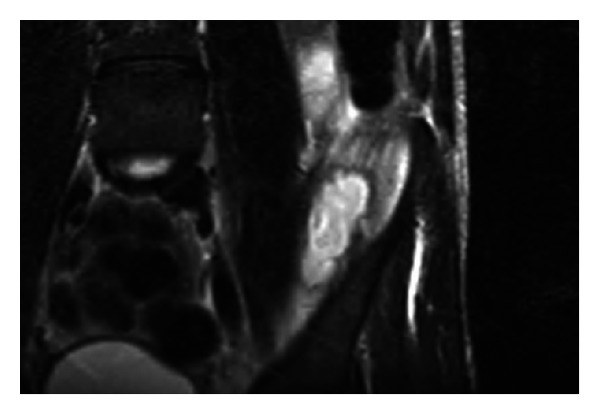
MRI image of iliacus abscess.

After washout, Mr. P.'s knee had improved significantly, however; the pain in his left sacroiliac joint and hip worsened. A whole-body MRI, before and after gadolinium, showed a left iliacus collection (length of 6 cm and a width of 3 cm—Figure 3), bilateral shoulder joint effusions, worse on the left, left knee joint effusions, and inflammatory change. Under sterile technique, a CT guided 14-French pig-tail catheter was placed via single pass into the left iliacus collection and remained in situ. A 25 mL of turbid thick pus was drained; microbiology was consistent with previous results. The bilateral shoulder collections were aspirated under ultrasound guidance and showed synovial blood-stained fluid with no frank pus identified, consistent with a reactive arthritis. A transthoracic echocardiogram showed no definite evidence of infective endocarditis and no visible vegetation.

A second arthroscopic washout was done 48 hours later, with no reaccumulation of pus seen. The patient remained haemodynamically stable and afebrile throughout admission. The iliacus drain was removed 6 days after insertion. 

The patient was discharged home 10 days after admission with a further 4-week course of IV flucloxacillin 12 g/24 hours via continuous Baxter pump infusion through a PICC line, before stepping down to oral flucloxacillin 1 g QID for a further two weeks. He was able to mobilise independently without walking aids. His left knee had 25 degrees of passive flexion and 5 degrees of passive extension at time of discharge. There were no further episodes of arthralgia/arthritis. He had full range of motion in all other joints. Subsequent blood cultures showed no growth, and inflammatory markers continued to improve. He was reviewed 9 days after discharge in the outpatient clinic, where his passive range of movement had improved to 40 degrees, and his inflammatory blood markers were within normal ranges. Mr. P. has finished his course of IV antibiotics and has made a complete recovery with full range of motion.

## 2. Discussion

A review of the literature on septic arthritis of the knee after ACL reconstruction demonstrates an infection rate of 0.3% to 1.7% [1, 2] with most infections within the first 2 months [2]. There have been no reported cases of haematogenous spread of infection from an iliacus abscess to an ACL reconstructed knee, let alone in a healthy young person with no risk factors. 

Kohn [3] reported the first case of a septic knee after arthroscopic-assisted ACL reconstruction in 1988. The greatest risk factor for the septic arthritis following ACL reconstruction has been found to be previous open knee surgery [4]. Other risk factors include the use of synthetic ACL grafts, intra-articular fixation and postoperative haemarthrosis [5, 6]. The incision for the tibial tunnel is the most common site for infection [7]. Mr. P. underwent ACL reconstruction with the use of an autologous hamstring graft, which has been found to have a lower infection rate compared to its synthetic counterparts. His tibial and femoral wounds had also healed unremarkably. From the literature, the mean time for infection after ACL reconstruction is 13.7 days. There has been no described infection of a hamstring reconstructed ACL knee 1 month after surgery [8, 9]. Mr. P. had two arthroscopic washouts of his knee. Both times, the knee was thoroughly irrigated and the graft carefully inspected. A survey taken from knee surgeons regarding their management of a septic ACL reconstructed knee showed that most would carefully inspect ACL graft and gently debride it on the first washout. The graft would only be removed if the signs and symptoms continue and if the graft looks obviously infected in the second washout [10, 11]. Since Mr. P.'s knee appeared clean on the second washout and the graft appeared intact, it was spared, and a decision was made to manage him with antibiotics and regular inflammatory marker monitoring.

Mr. P. initially presented with a history of vague abdominal pain for 48 hours prior to the knee pain. A review of the current literature has demonstrated no cases of haematogenous spread of infection from the iliacus abscess to an ACL reconstructed knee (or a native knee for that matter). Similar to a septic knee, *Staphylococcus aureus* is the causative organism in over 88% of patients with primary iliacus abscess [12]. In this case, *Staphylococcus aureus* was cultured from the iliacus abscess, knee, and blood cultures. On review, Mr. P. has no risk factors for an iliacus abscess. Iliacus abscesses have been associated with immunosuppression, autoimmune disorders, and long-term systemic disorders [13]. The principals of treatment are the evacuation of the collection and appropriate antibiotic cover. In this case, clinical recovery was only observed once the iliacus abscess was drained using a pig-tail catheter. Repeat imaging was conducted to confirm that the size of the collection was decreasing. 

## 3. Conclusion

This is an interesting case as there are several infective processes at play in this patient. Addressing only the septic knee would have led to recurrent infections and incomplete resolution of the infection. Through the use of urgent full body MRIs, we were able to identify other sources of infection and manage them accordingly. 

As described in the literature, ACL grafts can be salvaged from septic knees. We have described a case in which haematogenous spread of infection to an ACL reconstructed knee was successfully treated with full recovery and survival of the graft.

## References

[1] Schollin-Borg M, Michaelsson K, Rahme H (2003). Presentation, outcome, and cause of septic arthritis after anterior cruciate ligament reconstruction: a case control study. *Arthroscopy*.

[2] Judd MAJD, Bottoni LTCC, Kim D, Burke CPTM, Hooker MAJS (2006). Infections following arthroscopic anterior cruciate ligament reconstruction. *Arthroscopy*.

[3] Kohn D (1988). Unsuccessful arthroscopic treatment of pyarthrosis following anterior cruciate ligament reconstruction. *Arthroscopy*.

[4] Margheritini F, Camillieri G, Mancini L, Mariani PP (2001). C-reactive protein and erythrocyte sedimentation rate changes following arthroscopically assisted anterior cruciate ligament reconstruction. *Knee Surgery, Sports Traumatology, Arthroscopy*.

[5] Azar FM, Arthur ST (2004). Complications of anterior cruciate ligament reconstruction. *Techniques in Knee Surgery*.

[6] Pierre PS (2004). Complications of anterior cruciate ligament surgery. *Sports Medicine and Arthroscopy Review*.

[7] Binnet MS, Basahir K (2007). Risk and outcome of infection after different arthroscopic anterior cruciate ligament reconstruction techniques. *Arthroscopy*.

[8] Williams RJ, Laurencin CT, Warren RF, Speciale AC, Brause BD, O'Brien S (1997). Septic arthritis after arthroscopic anterior cruciate ligament reconstruction. Diagnosis and management. *American Journal of Sports Medicine*.

[9] McAllister DR, Parker RD, Cooper AE, Recht MP, Abate J (1999). Outcomes of postoperative septic arthritis after anterior cruciate ligament reconstruction. *American Journal of Sports Medicine*.

[10] Matava MJ, Evans TA, Wright RW, Shively RA (1998). Septic arthritis of the knee following anterior cruciate ligament reconstruction: results of a survey of sports medicine fellowship directors. *Arthroscopy*.

[11] Van Tongel A, Stuyck J, Bellemans J, Vandenneucker H (2007). Septic arthritis after arthroscopic anterior cruciate ligament reconstruction: a retrospective analysis of incidence, management and outcome. *American Journal of Sports Medicine*.

[12] Ricci MA, Rose FB, Meyer KK (1986). Pyogenic psoas abscess: worldwide variations in etiology. *World Journal of Surgery*.

[13] Mallick IH, Thoufeeq MH, Rajendran TP (2004). Iliopsoas abscesses. *Postgraduate Medical Journal*.

